# Older Adults’ Engagement in Residential Care: Pitfalls, Potentials, and the Role of ICTs

**DOI:** 10.3390/ijerph19052876

**Published:** 2022-03-01

**Authors:** Marije Blok, Barbara Groot, Johanna M. Huijg, Alice H. de Boer

**Affiliations:** 1 Leyden Academy on Vitality and Ageing, Rijnsburgerweg 10, 2333 AA Leiden, The Netherlands; groot@leydenacademy.nl (B.G.); huijg@leydenacademy.nl (J.M.H.); 2 Faculty of Social Sciences, Vrije Universiteit Amsterdam, de Boelelaan 1081, 1081 HV Amsterdam, The Netherlands; a.de.boer@scp.nl; 3 Department Public Health Eerstelijnsgeneeskunde, Leiden University Medical Center, Albinusdreef 2, 2333 ZA Leiden, The Netherlands; 4 SCP, The Netherlands Institute of Social Sciences, Bezuidenhoutseweg 30, 2594 AV The Hague, The Netherlands

**Keywords:** engagement, residential care, long-term care, older adults, ICTs

## Abstract

Over the previous years, the residential care sector has gone through a transition from a rather paternalistic approach towards a more democratic way of caregiving. Nevertheless, many care organizations still find it challenging to engage their residents in the process of care. In this study, we investigated the challenges regarding the engagement of older adults in residential care. As recent studies indicated the increasing opportunities of ICTs, we paid particular attention to this in the process of engagement. We followed a participatory action research approach among caregivers and older adults at a somatic care department in a care residence in the Netherlands. Methods used included 15 participants in two homogeneous group sessions, reflections on action in practice, and one mixed focus group. Our findings show that both caregivers and older adults acknowledge the importance of engagement in daily care. However, their different perspectives on how this should take place, made the actual engagement of older adults a challenge. We determined three dilemmas complicating this engagement in care, and labeled these (1) autonomy versus dependence; (2) personal experiences versus privacy; and (3) happiness versus honesty. We found different ways of how caregivers and older adults deal with these dilemma’s in practice and defined these in terms of pitfalls and potentials. ICTs were shown to reinforce both the pitfalls and potentials. Paying attention to these challenges in residential care, including how caregivers and older adults deal with these challenges, will encourage a mutual understanding and actual engagement in decisions on daily care. Further research is recommended on the role of organizations’ management, older adults’ relatives, or older adults with cognitive impairments.

## 1. Introduction

In recent years, the residential care sector has experienced a transition from a traditional, rather paternalistic approach focused on physical health, protocols, and standards towards a more democratic way of care, emphasizing relationships and person-centered care [1]. According to the concept of democratic care, quality of care can only be defined within the relationship between caregiver and care receiver [2,3]. This requires a continuous process of conversation, deliberation, and reflection in which the caregiver and care receiver together give substance to what good care includes. In line with this, Dutch care organizations have the legal obligation to establish a client council, based on the idea that decisions of the board should take into account the experiences with daily care as expressed by clients [4]. This is an encouraging trend, as several studies show a positive impact on older adults’ physical, social, and mental wellbeing from being engaged in the care they receive [5,6]. In practice, however, the establishment of a client council does not necessarily lead to collaborative decision making between caregivers and their clients [7].

Despite the fact that most caregivers considered providing care based on older adults’ needs and wishes as an important motivation to start working in the care sector [8], many of them still consider it a challenge to involve the older adults’ perspective in their daily care routine [9]. Previous studies showed, for example, the differences between caregivers and older adults in how they define wellbeing or understand engagement [10]. This disagreement did not appear among colleagues, as well as among older adults. A number of studies also have found that older residents experience limited opportunities to engage in activities in their residential setting [11]. Research on palliative care, e.g., indicated that older adults perceived that their mental health was underestimated by their caregivers [12].

In this study, we aim to explore why—despite good intentions—caregivers still face challenges in engaging older adults in the daily care process. Our research question is: ‘What challenges are faced in residential care when it comes to the engagement of older adults?*’* As older adults spend their day-to-day lives in this setting, we used a broad scope of care, including all kind of everyday experiences in the residence. In the literature several terms are used to indicate the older adults’ engagement. The terms ‘involvement’ and ‘engagement’ are most often used interchangeably [13]. In our study we will use the concept of older adults’ engagement. Inspired by research of Nierse, we define this as ‘decision-making within the care situation being carried out in collaboration with older adults rather than to, about, or for them’ [14].

In the collaboration between caregivers and older adults, information and communication technologies (ICTs) increasingly offer opportunities. Earlier research among older adults with (cognitive) impairments showed that ICTs can contribute to processes of social interaction, or to the perception of autonomy [15]. Recent research has identified a variety of ICTs that may support interaction with and engagement of older adults in the residential care setting [16,17]. In this study, we explore the challenges of older adults’ engagement. Considering the described increasing opportunities of ICTs, however, we additionally pay attention to the role of ICTs in this process. In line with earlier studies on this topic, we defined ICTs as all types of information and communication technologies used in order to gain or share information or to communicate with others, either face-to-face or at a distance [15]. This may include software or hardware, and could include both structured methodologies or daily use of own means. Studying this may deliver us handles for facilitating older adults’ engagement in a futureproof way.

## 2. Materials and Methods

### 2.1. Study Design

We followed a participatory action research (PAR) approach [18,19], including caregivers and older adults in a care residence. We considered this a suitable design, as within this approach the people studied conduct a study together with academics and other stakeholders to make a difference in their own lives. Instead of organizing activities particularly aiming at gaining knowledge, we joined and studied existing activities. This allowed us to learn in and from the real context, make impact, and limit the required time investment for participants. Characteristics of PAR are that the research process is collective, locally situated, participatory, and with a focus on transformation and impact. In PAR, all scales of impact have equal value. Personal change—such as changes in confidence, knowledge, and action in an individual—is, e.g., seen as equal to, and a necessary attribute of, other forms of change. Without personal change, wider impact is less likely to occur in a meaningful way [19]. Following a PAR approach, we did not only answer, but also formulated research questions in close collaboration with relevant actors. This required an open and flexible attitude from our participants but offered potential to really respond to the needs expressed, consequently increasing the likelihood of the eventual implementation of outcomes in practice.

### 2.2. Sample/Setting

We conducted this study in 2021 in a care home in a city in the Netherlands. Our research activities took place in a somatic care department (N = 20 caregivers; N = 25 residents, age 67–98;) in close collaboration with caregivers and older adults affiliated to this unit, as this department fitted properly to the research question we had. Older adults living in the department suffered from physical impairments, such as stroke, the lung disease chronic obstructive pulmonary disease (COPD), or the muscle disease amyotrophic lateral sclerosis (ALS). In this care residence, person-centered care was described in the policy as an important starting point for providing care and translated into approaches for caregivers. First, caregivers become acquainted with new residents by creating a Doodle Board together [20]. By means of a conversation, following a fixed structure, the caregiver learns more about the resident’s background. The output of this conversation serves as input for a creative poster to hang on the resident’s wall as a conversation piece. Second, throughout the day caregivers capture so-called experiences in SenseMaker^®^ [21]. With this mobile app, staff members describe a (care) situation, answer some reflective questions, and add an image of the situation. As a part of the job routine, these captured experiences are discussed among staff members. Although the concept of person-centered care was operationalized and implemented by these particular practices, we did not only focus on these methodologies. Instead we were interested in practices related to engagement in the residential setting in its broadest sense.

### 2.3. Research Team

The first author, M.B., researcher in the domain of meaningful ageing, conducted the fieldwork with a co-researcher whose function is that of mental caregiver in the care residence and an important linchpin between the involved caregivers and older adults. He was able to help us identify the older adults’ perspectives given his close relationship with them without being a regular caregiver; moreover, he was able to represent the caregivers’ perspective, being one of their colleagues. All activities throughout the study were defined, planned, and evaluated in collaboration with this mental caregiver. The other authors are critical friends (B.G. and J.M.H.) and the supervisor (A.H.d.B.) of the first author.

### 2.4. Research Activities

In line with the PAR approach, we followed a continuous cycle of action and reflection, conducting activities in an iterative way: the output of each step served as input for the next. Aiming to understand the challenges, we started investigating the perspectives of the stakeholders on the older adults’ engagement; we continued with experimenting with several new practices. Next, we describe the activities we conducted to meet our aims. Table 1 shows a compact overview of the activities as well as how the outcome of the one activity informed the set-up of the next one.

#### 2.4.1. Conversation Group with Older Adults

As a first activity to explore the older adults’ perspective on their own engagement in care, M.B. joined the residents’ conversation group in the department. This group meets weekly in variable size and composition for one-and-a half hours in the departments’ living room to exchange experiences about daily life in the care home. All older adults who were normally part of this group and were interested in and able to join our study, were welcome. Topics usually include practical issues such as dinner and bedtimes, as well as more fundamental issues, such as dealing with the loss of fellow residents. M.B. was invited to lead one of the sessions, which was attended by six older adults (four women, two men), one relative (woman) of an older lady, and a mental caregiver (man), who normally leads this group. In this group, we discussed with the older adults what was important to them in their daily residential life, how they would like to be engaged in these topics, and how they perceived current practices. Whereas we started with open questions about how the older adults preferred to be engaged in daily care, the session continued with the actual discussion of relevant themes.

#### 2.4.2. Pair Interview with Caregivers

As a substantial part of the output from the older adults appeared to be about their relationships and alignment with their caregivers, both the participants and researchers considered it useful to discuss these findings with caregivers as a next step. We invited three caregivers (one man, two women), but due to the daily concerns in care, we eventually carried out the session with two (women). In a dynamic pair interview, we explored caregivers’ perspective on the older adults’ engagement, discussed the older adults’ perspectives, and reflected on current practices. This allowed us to discover the similarities and differences between the perspectives of both types of stakeholders. Additionally, the caregivers proposed next steps to explore new practices, including a collaborative capturing of experiences and a mixed group session.

#### 2.4.3. Collaborative Action: Capturing Experiences Together

As a response to the caregivers’ proposal, we asked them to collaborate with an older adult in capturing meaningful experiences. Aiming to prepare the input for a mixed group session, the pairs were asked to capture, with image and text description, in Sensemaker^®^ [21], a number of daily experiences, through a period of two weeks. Eventually, two caregivers collected three experiences with three different residents. These experiences were not analyzed as such but served as input for the group session.

#### 2.4.4. Mixed Group Session with Caregivers and Older Adults

We organized a mixed group session with caregivers and older adults to facilitate the dialogue on the topics we had pinpointed thus far. This mixed session was attended by different types of caregivers (N = 5, including the mental caregiver, three general caregivers, one activity supervisor; three of them also participated in the previous research activities) and older adults (N = 10, including seven women and three men; six of them also participated in previous activities). We used the experiences captured by the pairs between caregiver and older adults as a basis for the conversation.

### 2.5. Data Analysis

We analyzed our data in an interactive and iterative way, following a thematic analysis approach [22]. After each step of data collection, we transcribed the audio footage verbatim. Thereafter, M.B. coded the transcript focusing on the relevant patterns in perspectives on the engagement of older adults. Important topics regarding the older adults’ engagement in received care were marked. Additionally, we paid attention to the use of ICTs throughout this process. As introduced earlier, this could include any type of information and communication technologies participants used in order to gain or share information or to communicate with others, either face-to-face or at a distance. This could, therefore, include specific applications such as the SenseMaker^®^ app, as well as daily technologies such as a smartphone or social media. After each step an infographic summarizing the key results was shared and discussed with the participants and co-authors. The next steps were defined together with the mental caregiver as a co-researcher.

### 2.6. Ethical Considerations

The data collection in this study was part of a broader study, which was reviewed and declared not subject to the law on research involving human subjects by the Institutional Review Board of the Medical Ethical Committee Leiden–Den Haag–Delft for observational studies, and registered under number N20.095. The protocol was assessed and considered compliant with scientific due diligence.

In line with this protocol, caregivers were informed and asked to sign an informed consent, in which they gave us their permission to use their input. In addition, we asked all participants of the three group sessions for their permission to (audio) record these. When transcribing the audio files, we used codes to indicate the participants to make them untraceable. Pictures taken throughout the study were used with permission of both those shown in the image and those who had taken the picture. The printed pictures were collected and destroyed by the researchers afterwards.

## 3. Results

Throughout the activities with our participants, we observed several challenges; although the caregivers and older adults expressed similar values to be important, they had different perspectives on them. We defined three key dilemmas when it came to the engagement of older adults in the care process, including:

(1) Autonomy versus dependence;
(2) Personal experiences versus privacy;
(3) Happiness versus honesty.

Participants provided several examples of how they dealt with these dilemmas in practice, either obstructing or supporting the successful engagement of older adults. We formulated these examples in terms of pitfalls and potentials. Table 2 provides an overview of the dilemmas, pitfalls, and potentials, as well as the role of ICTs.

### 3.1. Autonomy versus Dependence

#### 3.1.1. Perspectives

Both caregivers and older adults considered the perception of autonomy essential. At the same time, they described mutual dependence as inevitable in a residential care setting. Besides, caregivers and older adults did not always have similar perceptions and expectations of autonomy and dependence. This leaded to frustration, disappointment, or despondence between the two.

The older adults shared their perceived lack of autonomy across a range of topics, such as the time for serving dinner, people entering their room without asking, or the restrictions on playing games with fellow residents. This gentleman indicated the perception of daily decisions being made by caregivers one of the most difficult parts of residential life:

*“I’ve always been independent, but I came into a care home and got a completely different life. Then you no longer determine what happens. You’re not free anymore, you lose control over your own life. If you live normally, I mean, not in a care home, you decide everything yourself, what you do and don’t. That’s not the case here.”*

Caregivers recognized the lack of autonomy as perceived by the older adults. However, they indicated that their extent of control over the older adults’ daily lives was considerably overestimated. Instead, caregivers described that their autonomy was restricted by aspects such as organizational policy, staff capacities, restricted budgets, or older adults’ physical or cognitive conditions:

*“We do have to do it with the resources we have. From me they [the residents] can do anything, really. I want to move heaven and earth, but I have to do it with what my manager provides to me. If he says, ‘You have three evening shifts and one living room support person,’ I don’t have the control that the client can go to bed at the preferred time. or pee one more time. I want to, but I just can’t. Yes... that is very difficult.”*

#### 3.1.2. Pitfalls and Potentials

In our activities, participants shared examples of how they dealt with the dilemma between autonomy and dependence in practice, as well as how this obstructed or supported the older adults’ engagement. We found pitfalls, including patronizing and tokenism, as well as a potential, including shared power.

We found several examples in which caregivers seemed to underestimate older adults’ abilities to engage in the care process. We labeled this pitfall *patronizing*. Caregivers mentioned that due to cognitive impairments, older adults’ preferences keep changing over time, making it difficult for caregivers to understand what really matters to them. Although they found it important to take older adults’ input into account, they admitted that in such cases they decided to bypass their preferences and instead make decisions themselves; this often obstructed the successful engagement of the older adults in the care situation. Caregivers shared their doubts about the older adults being able to provide input for collaborative decision making on residential life. They suggested experimenting with triggering the older adults with pictures of daily situations to trigger them to provide input. When we shared some pictures taken by caregivers in the older adults’ focus group, to explore whether these would support them in sharing their input, older adults indicated that the pictures did not reflect their perception of daily life in the care home. Instead of feeling engaged in the process, the older adults perceived another loss of autonomy when they realized caregivers took pictures of them without their knowledge. As a response, we proposed to the older adults that they could take their own pictures to share their experiences with daily care. Although they were open to this idea, caregivers, in their own focus group, shared their skepticism of the older adults being able to do take pictures of care situations:

*“Clients can’t do that at all’,* [moves hands, to demonstrate motoric limitations] *they need assistance with that. I don’t even know if they have a mobile phone. They don’t take pictures, they don’t do that.”*

Also when it came to reflecting on daily care, caregivers seemed to underestimate older adults’ abilities. One of the caregivers described that one of her colleagues reported the perception of a lady without verification in the mobile app, she had also copied and attributed this reflection to all other residents:

*“After the movie night, for every resident she wrote, ‘Madam enjoyed the movie night.’ But every time. She just copied and pasted that. So I said to the resident, ‘I read that you had a movie night’. ‘Horrible,’ she says, ‘Someone next to me was drooling. Someone eyes closed’. Then I think, ‘Madam enjoyed the movie night..? Yeah…right.’”*

These examples show that the pitfall of patronizing can be reinforced by the use of ICTs. Although older adults shared their willingness to try out new things with their smartphones, such as taking pictures to share their perspectives, caregivers had little trust in their abilities and instead took over the control. This decreased their perception of being engaged.

We also found examples of situations in which input from the older adults was asked but, admittedly, not taken into account in the decision making. We labeled this pitfall *tokenism* [23]. For example, the caregivers in our study indicated that they found it important to provide an infrastructure supporting older adults to share their experiences with care. The conversation group we visited was one such a vehicle; the so-called living room meetings, organized for consulting the older adults, was another. The older adults, however, emphasized their input was rarely being followed up. Instead of feeling engaged in care, this lady described, they felt like their opinion was not considered important:

*“It is important that something is done with it. That it can be improved in some way. We have talked so many times about food. But nothing is done about it, nothing is improved. Nothing.”*

Additionally we observed situations in which older adults and caregivers together defined what good care included, as a successful way of engagement. We labeled this way of dealing with the dilemma *shared power*. As described, although the older adults shared their dissatisfaction with caregivers taking pictures and determining meaningful moments, they were not eager to capture experiences all by themselves. We therefore experimented with older adults and caregivers working together. We asked them, in pairs, to capture everyday meaningful moments in the SenseMaker^®^ app; i.e., uploading a picture, writing a description, and answering some additional questions [21]. In this way three experiences were collected collaboratively, including one of a caregiver and an older man getting to know each other in a personal conversation, one of an older man reminiscing about his time in the army back in the days, and one of an older man enjoying a herring for lunch. In contrast to pictures taken by caregivers, as discussed earlier, the older adults now were able to bring in what was important to them. Although the older adults found it difficult to reflect on the process, the captured experiences showed their contribution: they consciously posed for the picture and their experiences were captured with their literal quotes. The caregivers indicated this activity supported them in spending quality time with the residents and learning more about their experiences.

By focusing on the process of capturing experiences together rather than on the actual output, both older adults and caregivers could play a role. The use of ICTs turned out to enhance this potential. The SenseMaker^®^ app turned out to provide possibilities for this, guiding the users through the steps of capturing experiences. One of the pairs did not use the app at all, but instead only took and discussed a picture with the mobile phone of the caregiver. This turned out to be useful as well.

### 3.2. Personal Experiences versus Privacy

#### 3.2.1. Perspectives

Caregivers as well as the older adults expressed their need for personal contact in the collaboration on the care process. This caregiver indicated that knowing the older adults on a personal level, allowed her to provide care in a person-centered way:

*“I started talking to this gentleman, I communicated with him. I hadn’t done that before. I sat down next to him, in his room, and with the help of pictures we came to a very nice conversation. I chatted with him for an hour and I got to know him so differently. He is completely in my heart now, he is a beautiful man. [..] And now I think we were doing him a great disservice, this man. We have to do something with that.”*

Although older adults wished for care adjusted to their preferences, they were also fond of their privacy. Some of them described bad experiences with personal information being passed on, while others indicated they preferred to make a new start rather than keep talking about their past. Rather than introducing themselves through a personal conversation as part of their onboarding process, older adults rather gradually disclosed information about themselves, deciding for themselves what to share, to whom, and when. This lady explained this:

*“We have a cozy table, not a bad word to say. But you’re not going to say, ‘And madam, have you been married? Was your marriage good? Ah... did your husband die?’ We don’t talk about that. And I don’t think it’s necessary either. You can have a good time together... and if you want to say, ‘Well my husband...’ or whatever. Then it’s different, you build that up. If you treat each other normally, it comes up organically, right?”*

#### 3.2.2. Pitfalls and Potentials

Throughout our study, caregivers and older adults shared how they, as well as the residence’s management, dealt with the dilemma between sharing personal experiences and privacy in practice. We found pitfalls, including self-disclosure as a policy and privacy policy, as well as a potential, including reciprocity.

We found a number of situations in which getting to know the older adults on a personal level had become more of a goal in itself than spring from genuine interest. We labeled this *self-disclosure as a policy*. When a higher level of sharing personal information, in other words, self-disclosure, was asked from older adults than what they would prefer, this could have the opposite effect than intended [24]. According to this caregiver, this sometimes was at the cost of the older adults’ perceived wellbeing:

*“I’m getting to know a lady for the Doodle Board and she says, ‘It stresses me out.’ She also says: ‘It keeps me awake. I see it as a burden, what do you want to know?’ So I’ve really had a discussion [with manager], a couple of times already. ‘What do we want?’ ‘Yeah, we’re going to keep going anyway.’ It’s been decided that this should be done here.”*

We also observed situations in which the privacy of older adults was not taken into account when personal experiences were shared. We labeled this trend a lack of *privacy policy*. Whereas there was much attention to the policy for becoming personally acquainted with the older adults, the policy on how to deal with collected information was often lacking. A lack of policy on privacy can be considered a pitfall as it made older adults with low trust more reluctant, which obstructed their successful engagement. When we asked a caregiver whether they understood that older adults felt like their photos were being advertised, she responded:

*“Yes, of course. But you know what it is with social media. That’s come up very quickly and we’ve indicated to the management on a number of occasions that there actually needs to be a policy on that. Like, hey, you come to live here, permission is asked for photos. Those photos are used on social channels, to promote how, yes…”*

While several older adults already shared concerns about their input being passed on in face-to-face interaction, this distrust only increased with the possibilities of ICTs. ICTs offer countless potentials for recording and sharing situations with acquaintances and strangers. Participants mentioned examples varying from caregivers sharing digital pictures with each other using WhatsApp, printing photographs and displaying them on the corridors, to sharing pictures on social media, such as Instagram and Facebook.

Participants also shared examples of older adults and caregivers having a mutual dialogue. We labeled these examples *reciprocity* and considered this a potential for successful engagement of the older adult. This caregiver emphasized that her wish is not only to elicit personal information from residents, but instead have a reciprocal conversation:

*“Our goal is just to get to know each other better, because in the conversation of course I also tell a little bit about myself. Because it’s not a one-sided... um... direction.*

This wish for reciprocity is in line with what we know from earlier studies about self-disclosure, which show that intimacy will mainly be improved when both conversation partners have space to share something about themselves [25]. Reciprocity was also found to be a potential on a group level. In a mixed group session, we used the experiences that were captured by the pairs as a basis for the conversation. We printed the uploaded experiences—pictures and descriptions—beforehand. The pictures turned out to be useful for triggering an equal dialogue in which the different perspectives could be shared. Unfortunately, due to auditory and cognitive impairments, the gentlemen who participated in capturing the experiences were not able to attend the group session. Therefore, the caregivers shared the experiences on their behalf. Although we had aimed for them to be present, this situation unexpectedly showed us another added value of this practice: it allowed residents to learn more about their fellow residents who had mistakenly been seen as non-social. Capturing the experience allowed the absent residents to still be represented, this conversation in the mixed group session clarifies:

Lady: *“That man always walks down the hallway here.”*

Caregiver: *“Yes, he exercises himself.”*

Lady: *“And I’m just jealous of that, that man. But he doesn’t say hello. You can’t say: ‘Sir how well you move’ because he doesn’t say anything back.”*

Caregiver: *“Yes, but he doesn’t hear. That’s the problem.”*

The use of ICTs turned out to provide opportunities for preparing group dynamics, such as the mixed group session we organized. The use of ICTs by the participants for capturing their experiences before the group session provided additional potential for giving older adults a say who were otherwise limited in their ability to participate in social processes.

### 3.3. Happiness versus Honesty

#### 3.3.1. Perspectives

Both caregivers and older adults indicated the awareness of moments of happiness as an important precondition for continuing in residential life. This conversation between a lady and her caregiver illustrates this:

Lady: *“I’ve had a beautiful life, but it’s purposeless now.”*

Caregiver: ***“****We can take steps if we have a completed life.”*

Lady: ***“****No, I wouldn’t do that.”*

Caregiver: *Then what keeps you here?”*

Lady: ***“****There are moments of happiness anyway.”*

As moments of happiness were considered important, engaging older adults in care decision would imply the exploration of what makes them happy. Caregivers described that the older adults often find it difficult to define moments of happiness. According to the older adults, however, this is not necessarily a problem, as residential life should not always be about happiness. This lady shared:

*“You know what I’m sick of? That I have to like everything. It has to be nice at the table, there has to be music in the living room, we have to talk to each other. And you guys are just acting a little crazy and we have to find that funny. You know what I want? I don’t want anything. I don’t want anything, I don’t need anything. I’m in a rest home, I want peace and quiet. Stop being so nice and overenthusiastic.”*

After all, also caregivers indicated that strong focus on the positive side of life may easily lead to a denial of pain and sadness, which is often the honest reality in the care residence home. According to this caregiver, being honest would eventually help to accept life and its limitations in this stage:

*“I think it’s a good principle to focus on positive things and making residents happy. But I think you also have to be realistic and honest: ‘‘I’m never going to make someone feel at home here.’ I think accepting the fact that nobody will feel at home here, helps in your own frustration. Be realistic, that’s quite important.”*

#### 3.3.2. Pitfalls and Potentials

Our participants shared several examples of how they dealt with the dilemma between a focus on happiness on the one hand and on the honesty on the other hand. We found a pitfall, namely the use of moments of happiness as a marketing instrument, as well as potential, including shared sense-making.

Participants shared several situations in which the care residence was presented as a holiday resort to attract prospective residents and their relatives. We labeled this pattern the use of moments of happiness as a *marketing* instrument. This positive presentation was done, for example, on the organization’s website, in brochures, or during an introduction tour. Participants considered this a pitfall as it, first, does not prepare new residents honestly for their stay in the residence and, second, current residents do not feel represented properly, which obstructs successful engagement of the older adults. A caregiver shared:

*“They* [management] *want to sell it* [care home] *here as a kind of holiday resort. That is the trend. Own control, enough activities! But what is the reality? A lot of sadness.”*

We observed this positive way of presenting life in the care residence also at a more individual level, such as in communications to residents’ relatives, reassure them that their beloved ones are enjoying their stay. Although caregivers found it important to be honest, in their opinion, it was not always constructive to share all details of complicated care situations with family:

*“But, what matters to me … when I say, ‘Madam is enjoying an activity’, it’s also because the family reads that. There is already so little reporting, you know, and it’s actually always about all the physical and medical things.”*

Like we mentioned earlier, ICTs offer countless potentials for recording and sharing experiences. Additional to privacy issues, this is also a concern with regard to the pitfall of marketing. This caregivers indicated that they found social media usually not appropriate to share the complicated things of the care situation, as users of such platforms rather see positive messages:

*“We started with Facebook at one time, with sharing things, because the organization asked us to: ‘Show what you are doing with your client. How extensive our range of activities is’. So you have to imagine, those activity leaders who faithfully post all that. You don’t put down: It was a shitty day, they didn’t like it, but I’m going to post some pictures because that’s what the higher-ups want. Fair is fair, so you don’t do that.”*

Participants also came up with examples of how daily life in the care residence could be represented in a positive, yet realistic way. We labeled this potential *shared sense-making*. We discussed with caregivers ways to engage the older adults and their relatives in an honest way, without worrying them. Instead of formulating the experience on your own, the process of giving meaning to moments should be done together, this caregiver emphasized:

*“Ask a resident how he perceived a situation. Afterwards. Anyway: just don’t post that picture right away without checking. Like: ‘madam enjoyed the movie night’, but just go a day later. ‘That movie night, what did you actually think of it?’”*

Additionally, caregivers mentioned that the trend of sharing pictures instead of written reports provides potential to share experiences without putting the older adults’ perception into words. Instead of struggling with what message to share, a picture itself may tell the word. As family members have known their loved ones for a long time, they may be even better in interpreting what they see. In this way, this potential not only provides opportunity for the engagement of the older adults but also with their relatives, this caregiver shared:

*“In the end, a photo like this is also a piece, hopefully, for the family. That they could see: ‘My mother has been in the garden’. Instead of that sentence, you see a picture of someone. Yeah, you know, that’s just...Then someone can interpret for themselves, ‘Well, my mother looks a little grumpy’ or, ‘Yes, that’s how I recognize my mother, yes that’s how she used to sit.”*

ICTs offer opportunities to mediate sensory triggers. This allows sharing experiences not only in written text also, e.g., in image, sound, or video. ICTs, in general, or the SenseMaker^®^ app more specifically, can support the process of shared sense-making, as it allows users to share experiences close to the reality, without immediately adding their own interpretation in words.

## 4. Discussion/Conclusions

In this study, we explored the challenges in the engagement of older adults in residential care. Throughout a participative and iterative process with caregivers and older adults, we discovered three key dilemmas, namely, autonomy versus dependence, personal experiences versus privacy, and happiness versus honesty. Although caregivers and older adults shared similar values to be important, their perspectives on these may be conflicting. For each dilemma, we described pitfalls and potentials, as well as how ICTs obstructed or supported successful engagement. Based on our findings, we draw the following conclusions:

### 4.1. Responding to the Challenges of Democratic Care

Our findings show that successful engagement of older adults in a care residence does not ask for a facilitating attitude or policy from caregivers allowing older adults to share their perspectives per se. It rather requires a routine that provides space for caregivers and older adults to collaborate continuously in a personal way, so that the older adults’ voices can be integrated in daily care. This is in line with the definition of democratic care, stating the importance of a continuous process of conversation, deliberation, and reflection [2,3]. However, with our study, we provide concrete handles, i.e., shared power, reciprocity, and shared sense making, for giving substance to this process. When utilizing the potentials, engagement of older adults goes beyond the level of ‘consultation’, like we often see in client councils, or like we observed in our activities. Instead, successful engagement is about evaluating the quality of care together and bringing it further. In this case, the issue of freedom of choice regarding bedtimes is a typical example, which older adults worldwide, such as the participants in our study, are asking for [26]. When the process engagement is integrated in a proper way, a better understanding between caregivers and older adults will be reached and the right themes will be worked on [27].

### 4.2. Pitfalls and Potentials of ICTs

When it came to the use of ICTs, we found that deployment of these reinforced the discovered pitfalls and potentials regarding the older adults’ engagement.

In this study, a first pitfall reflected in the use of ICTs was patronizing. Although residents seemed to be cautiously open to taking pictures with their smartphones, caregivers were not confident in their abilities. This is worrying, as earlier research showed that inhibiting older adults’ use of ICTs can reduce feelings of engagement and autonomy [13]. Another pitfall enhanced with the use of ICTs included the lack of privacy policy and the use of happy moments as a marketing instrument. While several older adults shared concerns about their input being passed on in face-to-face interaction, this distrust only increased with the possibilities of ICTs. ICTs offer countless potentials for recording and sharing situations with acquaintances and strangers. Dealing with this incorrectly, e.g., by not taking into account privacy issues, or by presenting the care residence in a too positive way, can threaten the relationship between caregivers and older adults. The concerns regarding privacy when using telehealth interventions were described in earlier research [28]. Our findings show, however, that digital applications are not only used as particular interventions but are part of daily life nowadays. If we would like to promote the engagement of the older adults, agreements on privacy issues also should be defined in an interactive way, rather than as an informed consent provided by the care organization.

The first potential that could be enhanced with ICTs was shared power. By focusing on the process of capturing experiences together rather than on the output, both caregivers and residents could play a fair role. ICTs proved to offer possibilities for this. Another potential reinforced by the use of ICTs included reciprocity. In engaging older adults with impairments equally in a reciprocal conversation, ICTs appeared to play a supportive role. The use of ICTs for capturing experiences allowed caregivers to represent residents who were otherwise limited in their ability to participate in social processes. This type of deployment of ICTs can be considered a compensation strategy, as the representation of residents in the conversation group by pictures compensated for the disability of participating in real life. This is in line with theories on the mechanism of compensatory behavior as people age [29] and other studies on ICTs that show the potentials of ICTs for such compensatory behavior [15]. However, the role of caregivers taking the initiative to take pictures and represent older adults in the group has not been extensively reported in previous research. The potential of shared sense-making was also enhanced by the use of ICTs. Defining good care in a democratic process requires tools with room for interpretation from different perspectives. Previous research described the potentials of ICTs to mediate different types of sensory triggers when exchanging. This allows exchanging experiences not only in text [30] but also, e.g., in image, sound, or video. ICTs in this way can support the process of shared sense-making as it allows users to share experiences close to the reality, without immediately adding interpretation in words.

Although we used the SenseMaker^®^ app as an application to capture experiences, we also observed participants to use their own smartphones. This is in line with one of our previous studies, in which we found that users of ICTs find their own ways of reaching their goals [15].

### 4.3. Strength, limitations, and Further Research

Following a PAR approach turned out to be a proper way to explore the challenges of older adults’ engagement in the residential care. Studying this setting in a participative way allowed us to work together with our participants on a long term. We could build up relationships with the caregivers and older adults and develop activities and research questions fitting within their preferences and abilities. Although we studied only one care residence, we were able to learn about the principles and practices that may be representative of other residences as well, working according to the principles of democratic care.

A significant finding from our study is the importance of the inclusion of different perspectives on engagement throughout the process of daily care. Instead of either the caregiver or the older adult giving meaning to the care situation, we should aim for making decisions in dialogue. We found ICTs to provide several opportunities for this. Further research is needed to examine this in a more systematic way, so the increasing potentials of ICTs can be utilized in the process of engagement. In this study, we only involved residents and several types of caregivers, which can be considered a limitation. We recommend further research to include other actors, such as policymakers, managers, and older adults’ relatives as well. We also restricted this study to the somatic care department. Further research is needed to learn more about our findings in the context of older adults with cognitive impairments.

Providing person-centered care is a major goal in care homes, but the successful implementation of this policy is associated with several dilemmas, as perspectives on engagement differ among older adults, caregivers, and other stakeholders in the residence. When dealing with these dilemmas, stakeholders tend to keep in their traditional roles, restricting successful engagement of older adults. When caregivers are conscious of this tendency, it improve the mutual understanding and successful engagement of older adults. In this paper, a first step was taken to provide insight into how the potential of ICTs can be exploited in such a way that it raises the engagement of older people in this setting.

## Figures and Tables

**Table 1:** Overview of the research activities.

Research Activity	Aim	Analysis of Output
Group session in regular conversation group setting with older adults (N = 10 older adults; 1 mental caregiver (co-researcher); 1 relative of older adult). t = 1.5 h)	Exploring older adults’ perspectives on engagement; Reflecting on aims and practices of the conversation group; Learning about how to trigger dialogues among older adults;	Transcription verbatim; Coding with focus on older adults’ needs and wishes, and their perception of current practices; Infographic, shared with participants and co-authors; → Next step based on results: reflecting on findings with caregivers;
Pair interview with professional caregivers (N = 2 (female) caregivers, one (male) caregiver applied but had to apologize last minute) t = 1.5 h);	Exploring caregivers’ perspectives on older adults’ engagement; Reflecting on older adults’ needs, wishes, and current practices; Defining dilemmas in current practices;	Transcription verbatim; Coding with focus on caregivers’ needs and wishes and perception of current practice; Infographic, shared with participants and co-authors; → Next step based on results: exploring caregivers and older adults working together;
Collaborative action; capturing experiences together	Exploring older adults’ involvement in capturing experiences; Exploring ICTs’ potentials in this process;	One-to-one reflection with caregiver in one-to-one and mixed group setting; → Next step based on results: discussing experiences with others in mixed group;
Mixed group session (N= 15) with caregivers and older adults to exchange experiences;	Discussing the potentials of sharing experiences in mixed group setting; Exploring ICTs’ potentials in this process;	Transcription verbatim; Coding with focus on experiences with new practice; Infographic, shared with participants and co-authors;

**Table 2:** Dilemmas, pitfalls, and potentials.

Dilemmas	Pitfalls	Potentials	The Use of ICTs
1. Autonomy versus dependence	Patronizing	Shared power	−Underestimating the abilities of older adults’ use of ICTs; +Using ICTs in a collaborative process between caregiver and older adult;
	Tokenism		
2. Personal experiences versus privacy	Self-disclosure as a policy	Reciprocity	−Using social media before protecting personal data; +Representing older adults with difficulties to participate;
	Privacy policy		
3. Happiness versus honesty	Marketing	Shared sense-making	−Using digital media to tell a success story about residence; +Utilizing the potentials of ICTs for displaying experiences and triggering senses;

## Data Availability

Data available on request due to privacy restrictions.
